# Taohong Siwu Decoction Combined With the LncRNA H19/miR-675-5p Axis Repairs Limb Ischemia-Reperfusion Injury Through the Regulation of the Wnt3a/Ca^2+^ Signaling Pathway

**DOI:** 10.1155/mi/3096848

**Published:** 2025-02-23

**Authors:** Fuping Zhu, Hui Liu, Yinsheng Cao, Bing Dai, Hang Wu, Yutong Zhu, Wuping Li

**Affiliations:** ^1^Department of Foot and Ankle Orthopedics, The First Hospital of Hunan University of Chinese Medicine, Changsha 410007, Hunan, China; ^2^Department of Orthopedic Surgery, The Second Xiangya Hospital of Central South University, Changsha 410011, Hunan, China; ^3^Department of Pharmacy, The First Hospital of Hunan University of Chinese Medicine, Changsha 410007, Hunan, China; ^4^The First Clinical College of Traditional Chinese Medicine, Hunan University of Chinese Medicine, Changsha 410208, Hunan, China

**Keywords:** limb ischemia-reperfusion injury, LncRNA H19/miR-675-5p axis, Taohong Siwu decoction, Wnt3a/Ca^2+^ signaling pathway

## Abstract

**Background:** Taohong Siwu decoction (THSWT) has shown therapeutic effects on ischemia/reperfusion injury (IRI). This study tended to investigate the role of THSWT combined with the long non-coding RNA (LncRNA) H19 (H19)/miR-675-5p axis in improving limb IRI (LIRI).

**Methods:** Hind LIRI rats and simulated IRI skeletal myoblasts models were constructed to evaluate the therapeutic effects of THSWT. The mechanism of THSWT treatment on LIRI was investigated by the regulation of the H19/miR-675-5p axis and the wingless/integrated (Wnt)/Ca^2+^ signaling pathway. Various assessments, such as H&E staining, TUNEL staining, flow cytometry, cell counting kit-8 (CCK-8) assay, quantitative real-time polymerase chain reaction (qRT-PCR), western blot, immunohistochemistry (IHC) staining, enzyme-linked immunosorbent assay (ELISA), biochemical assay, and calcium fluorescence imaging, were conducted to observe skeletal muscle injury, cell apoptosis, skeletal myoblast proliferation, gene and protein expressions, cytokine levels, glucose (Glu) uptake, and Ca^2+^ concentration.

**Results:** THSWT intervention effectively improved skeletal muscle injury in LIRI rats, as evidenced by reduced muscle fiber damage and decreased cell apoptosis, accompanied by downregulation of H19, miR-675-5p, cleaved-Caspase3, Bax, PLC, and PKC expressions and upregulation of Bcl2 expression. Furthermore, silencing of H19 inhibited cell apoptosis of skeletal muscle and reduced IL-1*β*, IL-6, and TNF-*α* levels in LIRI rats. Notably, THSWT intervention combined with the silencing of H19 synergistically promoted the repair of skeletal muscle injury in LIRI rats. Mechanistically, THSWT intervention combined with regulation of the H19/miR-675-5p axis promoted the proliferation of skeletal myoblasts damaged by IRI through the Wnt3a/Ca^2+^ signaling pathway, increasing the levels of intracellular Bcl2, while decreasing the levels of Ca^2+^, CaMKⅡ, PLC, PKC, cleaved-Caspase3, Bax, TNF-*α*, IL-1*β*, IL-6, Wnt3a, and *β*-catenin.

**Conclusions:** THSWT combined with the regulation of the H19/miR-675-5p axis effectively improved LIRI by modulating the Wnt3a/Ca^2+^ signaling pathway, providing insights for potential therapeutic strategies for LIRI.

## 1. Introduction

Limb ischemia/reperfusion injury (LIRI) refers to the cellular damage and further deterioration of limb function caused by the reintroduction of blood following a period of ischemia. The pathological mechanisms and processes of LIRI are complex and not yet fully understood [1]. LIRI involves multiple factors and cell types and may vary depending on the affected tissues and organs as well as the duration of tissue ischemia [2]. Compared to the extensive research on ischemia/reperfusion injury (IRI) in vital organs like the heart, liver, and brain, studies on LIRI in limbs are relatively limited. However, skeletal muscle, which is the main tissue in the limbs, is highly sensitive to ischemia and irreversible damage can occur after just 3 h of ischemia [3, 4]. Severe LIRI can lead to multiple organ dysfunction syndrome (MODS), which is a life-threatening condition for patients [5, 6]. Therefore, it is urgently necessary to seek effective therapeutic approaches for LIRI, which has become our top priority.

Long-term clinical practice and animal experimental studies have shown that traditional Chinese medicine (TCM) has unique advantages in IRI treatment. TCM theory focuses on understanding the pathological mechanisms of IRI, such as blood stasis and impaired blood circulation [7–10]. Taohong Siwu Decoction (THSWT), first mentioned in Wu Qian's “Yizongjinjian” during the Qing Dynasty, is a TCM classic formula composed of six medicinal herbs, including peach seed and safflower, Szechuan lovage rhizome, prepared Rehmannia root, *Angelica sinensis*, and red peony root [11]. Modern pharmacological research has shown that THSWT has multiple beneficial effects, such as improving abnormal blood rheology, inhibiting inflammatory cytokines, relieving microcirculation obstruction, and enhancing immune function [12]. Additionally, THSWT can exert therapeutic effects in various diseases through multiple mechanisms. For example, it can treat acute blood stasis in rats by regulating amino acid and lipid metabolism [13]. Studies have also shown that THSWT can reduce cerebral infarct volume and improve neurological function in stroke model rats by modulating the phosphatidylinositol 3-kinase/protein kinase B (PI3K/Akt) and nuclear factor erythroid 2-related factor 2 (Nrf2) signaling pathways [14]. Moreover, THSWT has remarkable effects in the treatment of bone injuries by regulating the vascular endothelial-derived growth factor-focal adhesion kinase (VEGF-FAK) and hypoxia-inducible factor-1*α* (HIF-1*α*) signaling pathways [15, 16]. Recently, research from the perspective of TCM on the mechanisms of treating various diseases has been deepening. Therefore, we are interested in studying the use of THSWT in combination with targeted therapies for the treatment of LIRI.

More and more evidence suggests that long non-coding RNA (LncRNA) and microRNA (miRNA) play crucial roles in IRI pathogenesis [17–19]. Studies have investigated the potential roles of H19 and its derived miR-675 in regulating myocardial IRI and have found that their expressions are upregulated in oxygen–glucose-deprivation/reperfusion (OGD/R) myocardial cells. Knocking down H19 can improve cell viability, reduce apoptosis, decrease levels of inflammatory cytokines, and inhibit oxidative stress [20]. Another study has revealed that H19 expression is upregulated, while miR-181a-5p expression is downregulated in spinal cord IRI (SCIRI) mice and OGD/R cells. Knocking down H19 promotes hind-limb motor function recovery and alleviates pathological damage, cell necrosis, and inflammation caused by SCIRI [21]. However, there is limited research on the role of H19 in LIRI, and the related regulatory mechanisms remain to be clarified, which is a novel aspect of this study. It has been found that hypoxia induces miR-675-5p expression, which can enhance the HIF-1*α* response and activate the wingless/integrated (Wnt)/*β*-catenin signaling [22]. Studies have indicated that maintaining Ca^2+^ homeostasis is crucial for muscle injury repair [23–25]. The Wnt/Ca^2+^ signaling pathway plays an important role in skeletal development and related diseases [26]. However, the regulatory mechanism of the Wnt/Ca^2+^ signaling pathway in skeletal muscle injury caused by lung IRI still requires further investigation.

Previous studies have shown that the classic Wnt3a ligand not only stimulates *β*-catenin transcriptional activity in colon cancer cells, but also activates PLC, promotes Ca^2+^ mobilization, and induces Rho kinase and PLC-dependent cell migration [27, 28]. The LncRNA AZIN1-AS1/miR-6838 axis inhibits cell apoptosis by activating the Wnt3a/*β*-catenin pathway, thereby, protecting against myocardial IRI [29]. Wnt3a is downregulated in both in vivo and in vitro models of brain IRI [30] and mediates blood–brain barrier damage related to cerebral IR [31]. The above studies confirm that Wnt3a mediates IRI, but its expression and role in LIRI still need further exploration. Based on the above findings, this study established a LIRI rat model and a simulated IRI skeletal myoblast model. It further explored the mechanism of skeletal muscle injury in LIRI and the intervention effect of THSWT from the perspective of the Wnt3a/Ca^2+^ signaling pathway. This study provided experimental evidence to enrich the understanding of the pathological mechanism and treatment methods of LIRI.

## 2. Materials and Methods

### 2.1. Preparation of THSWT

THSWT was manufactured at the Dispensing Department of the First Affiliated Hospital of Hunan University of Chinese Medicine using modern processing techniques. The formula consisted of peach seed and safflower, each weighing 15 g, and Szechuan lovage rhizome, prepared Rehmannia root, *A. sinensis*, and red peony root, each weighing 10 g. The preparation process for THSWT was determined based on the physicochemical properties of its active ingredients. The six herbs were extracted with water, and the resulting water extract was then concentrated under vacuum until it reached a relative density of 1.08–1.12, with a temperature of 60°C. Sodium benzoate was added to dissolve the concentrated solution, resulting in the final product, THSWT. The original concentration of THSWT was 1.75 kg/L.

### 2.2. Animal Model Construction and Intervention

Male Sprague-Dawley (SD, 8 weeks, 240–300 g) rats were bought from Hunan SJA Laboratory Animal Co., Ltd. (Changsha, China). All rats were housed in pathogen-free cages with six rats per cage. The conditions included a temperature of 25 ± 2°C, humidity of 50% ± 5%, and a 12-h light/dark cycle, with free access to food and water. After a 1-week acclimation period, the experiments began. The SD rats were randomly divided into eight groups: Normal, LIRI, THSWT, sh-NC, sh-H19, inhibitor NC, miR-675-5p inhibitor, and THSWT + miR-675-5p inhibitor, with six rats in each group. For the THSWT and THSWT + miR-675-5p inhibitor groups, rats were administered THSWT orally at a dose of 10 mL/kg, twice a day for three consecutive days before modeling. The remaining groups of rats were given an equal volume of normal saline by oral gavage. One more intervention was performed 2 h before molding. Additionally, rats in the sh-NC, sh-H19, inhibitor NC, miR-675-5p inhibitor, and THSWT + miR-675-5p inhibitor groups were injected with 50 nmol/L of sh-NC, sh-H19, inhibitor NC, and miR-675-5p inhibitor via the tail vein 2 h before modeling. sh-NC, sh-H19, inhibitor NC, and miR-675-5p inhibitor were obtained from Honorgene (Changsha, China). Except for the Normal group, the other groups of SD rats underwent LIRI modeling based on the previous experimental methods. Four hours after ischemia, the paw became pale, with local skin appearing blue and purple, and the skin temperature became cool. The movement of the affected limb was significantly limited. After reperfusion, the paw returned to a reddish color and warm temperature, indicating the successful construction of the LIRI model. Two hours after reperfusion, the rats were sacrificed using cervical dislocation. Samples of skeletal muscle were collected, fixed in 4% paraformaldehyde (N1012, NCM Biotech, China), embedded in paraffin, and then subjected to histological analysis. All animal experimental procedures were approved by Animal Experimental Ethical Inspection in the Laboratory of the First Hospital of Hunan University of Chinese Medicine (ZYFY20220418-2) and were conducted following the ARRIVE guidelines for reporting animal research.

### 2.3. Preparation of THSWT-Containing Serum

One hour after the last administration, SD rats in the THSWT group were anesthetized by intraperitoneal injection of 2% pentobarbital sodium solution (40 mg/kg). Blood samples were collected from the abdominal aorta. The collected blood was placed at 37°C for 1 h, followed by centrifugation at 3000 rpm for 10 min to separate the serum. The serum was then filtered through a 0.22 μm sterile membrane to obtain the THSWT-containing serum for cell experiments.

### 2.4. Preparation of Simulated IR Solution

The preparation of simulated IR solutions was referred to literatures [32, 33]. Preparation of simulated ischemic solution (units: mM): NaH_2_PO_4_: 0.9, NaHCO_3_: 6.0, CaCl_2_: 1.8, MgSO_4_: 1.2, sodium lactate: 40, HEPES: 20, NaCl: 98.5, KCl: 10.0, sodium metabisulfite: 10.0, pH: 6.8. Preparation of simulated reperfusion solution (units: mM): NaCl: 129.5, KCl: 5.0, NaH_2_PO_4_: 0.9, NaHCO_3_: 20, CaCl_2_: 1.8, MgSO_4_: 1.2, HEPES: 20, glucose (Glu): 55, pH: 7.4.

### 2.5. Cell Culture and Treatment

Skeletal myoblasts were isolated from the hind legs of normal SD rats and purified as described in our previous study [34]. They were cultured in DMEM (D5796, Sigma, USA) supplemented with 10% fetal bovine serum (FBS; 10099141, Gibco, USA) and 1% Penicillin/Streptomycin (SV30010, Beyotime, China), and maintained in a humidified incubator (DH-160I, SANTN, China) with 5% CO_2_ at 37°C. Passage and transfection experiments were conducted when the cells reached 70%–80% confluence. The three to six generations of skeletal myoblasts were used in the following experiments:

After the medium was removed, skeletal myoblasts were added with a simulated ischemic solution and incubated for 8 h. After aspiration of the simulated ischemic solution, skeletal myoblasts were incubated with simulated reperfusion solution for 3 h to establish a cell injury model. The cells were then divided into Control, si-NC, si-H19, si-H19 + mimic NC, si-H19 + miR-675-5p mimic, and si-H19 + miR-675-5p mimic + THSWT groups. si-NC, si-H19, mimic NC, and miR-675-5p mimic were purchased from Honorgene (Changsha, China). Except for the Control group, cells in the other groups were transfected with si-NC, si-H19, mimic NC, and miR-675-5p mimic using Lipofectamine 2000 (11668019, Invitrogen, USA). After transfection, cells in the si-H19 + miR-675-5p mimic + THSWT group were cultured in THSWT-containing serum.

### 2.6. H&E Staining

After being baked at 60°C for 12 h, skeletal muscle slices were dewaxed to water. Sequently, the slices were stained with hematoxylin (AWI0001a, Abiowell, China) for 1 min and rinsed with distilled water. After returning to blue with phosphate-buffered saline (PBS), they were stained with eosin (AWI0029a, Abiowell, China) for 1 min. Then, the slices were rinsed with distilled water and dehydrated with gradient alcohol (95%–100%). Further, the slices were immersed in xylene for 10 min to achieve transparency. Finally, they were sealed with neutral gum (AWI0238a, Abiowell, China) and observed under a microscope.

### 2.7. TUNEL Staining

The skeletal muscle slices were baked at 60°C for 12 h and then dewaxed to water. Testing for cell apoptosis was conducted using a TUNEL detection kit (40306ES50, Yeasen, China). The slices were treated with 100 μL of proteinase K solution at 37°C for 20 min, followed by 100 μL of equilibration buffer at 25°C for 30 min and finally, 50 μL of TDT enzyme solution at 37°C for 1 h. The slices were then stained using DAPI solution at 37°C for 10 min and secured with glycerol for future observation.

### 2.8. Quantitative Real-Time Polymerase Chain Reaction (qRT-PCR)

The total RNA was extracted from both skeletal muscle and skeletal myoblasts through the use of Trizol reagent (15596026, Thermo, USA), which was later processed into cDNA utilizing an mRNA reverse transcription kit (CW2569, CWBIO, China). Analysis of the gene expression was performed with the QuantStudio1 Real-Time PCR System (ABI, USA) by applying the UltraSYBR Mixture (CW2601, CWBIO, China). To assess the relative gene expression, the 2^−*ΔΔ*Ct^ method was utilized with GAPDH as a standard reference gene. The specific primers used are detailed in Table 1.

### 2.9. Western Blot

Total proteins were extracted from skeletal muscle and skeletal myoblasts using RIPA lysis buffer (AWB0136, Abiowell, China). The proteins were isolated by SDS-PAGE and transferred onto nitrocellulose (NC) membranes. The membranes were blocked with 5% skimmed milk (AWB0004, Abiowell, China) for 1.5 h and then incubated with primary antibodies at 4°C overnight. Subsequently, the membranes were incubated with secondary antibodies at room temperature for 1.5 h. Super ECL Plus detection reagent (AWB0005, Abiowell, China) was applied for chemiluminescence imaging (ChemiScope6100). The gray values of protein bands were analyzed by Quantity One 4.6.6 (Bio-Rad Inc., USA), and the protein expression was calculated with GAPDH as an internal reference. The information on antibodies is exhibited in Table 2.

### 2.10. Enzyme-Linked Immunosorbent Assay (ELISA)

Whole blood samples were placed at room temperature for 2 h and then centrifuged at 4°C at 1000 × *g* for 15 min. The cell cultures were centrifuged at 4°C at 1000 × *g* for 15 min to obtain the supernatant. IL-6 (CSB-E04640r, CUSABIO, China), IL-1*β* (CB-E08055r, CUSABIO, China), and TNF-*α* (CB-E11987r, CUSABIO, China) ELISA kits were applied to evaluate IL-6, IL-1*β*, and TNF-*α* levels. Glu (A154-1-1, Nanjing Jiancheng Bioengineering Research Institute) were applied to evaluate Glu level via the Glu oxidase method.

### 2.11. Immunohistochemistry (IHC)/Immunocytochemistry (ICC) Staining

For skeletal muscle, slices were baked at 60°C for 12 h and dewaxed to water. Then, the citrate buffer (0.01 M, pH 6.0; AWI0206a, Abiowell, China) was applied for antigen retrieval and 1% periodic acid was employed for endogenous enzyme inactivation. For skeletal myoblasts, the cell slides were washed three times with PBS. They were then fixed with 4% paraformaldehyde for 30 min. After that, 0.3% Triton X-100 was added to cell slides and incubated at 37°C for 30 min for permeabilization. Next, 3% H_2_O_2_ was added to deactivate endogenous enzymes for 10 min. Tissue slices were incubated with primary antibodies of PKC (1:200, ab124735, Abcam, UK) and PLC (1:200, ab181558, Abcam, UK) at 4°C overnight while cell slides were incubated with primary antibodies of *β*-catenin (1:200, 17565-1-AP, Proteintech, USA) under the same conditions. Tissue slices and cell slides were then incubated with 100 μL of HRP-goat anti-rabbit IgG (1:100, AWS0005, Abiowell, China) at 37°C for 30 min. Then 100 μL of DAB (ZLI-9018, ZSBG-Bio, China) was added and incubated for 5 min for color development. Slices were restained with hematoxylin for 5 min, rinsed with distilled water, and returned to blue with PBS. Slices were dehydrated with gradient alcohol (60%–100%) for 5 min per level. The slices were placed in xylene for 10 min for transparency for future observation after being sealed with neutral gum.

### 2.12. Calcium Fluorescence Imaging

Fluo-3 AM (S10561056, Beyotime, China) was diluted to 5 μM in serum-free medium at 1:1000. Then, an appropriate volume of diluted Fluo-3 AM solution was added to the medium-removed cells. The volume of the addition should be sufficient to cover the cells. The cells underwent a 30-min incubation at 37°C in a humidified incubator. To eliminate any Fluo-3 AM that had not penetrated, the cells were rinsed thrice with a serum-free medium. A fluorescence microscope was used to check the fluorescence status of each group and photos were taken.

### 2.13. Cell Counting Kit-8 (CCK-8) Assay

After treating skeletal myoblasts with 0.25% trypsin, they were placed into 96-well plates at a concentration of 5 × 10^3^ cells/well. Following cell adhesion, appropriate treatments were given. Subsequently, the initial medium was replaced with 100 μL of medium that included 10% CCK-8 reagent (NU679, DOJINDO, Japan). After incubating these cells at 37°C for another 4 h, the optical density (OD) at 450 nm was measured using a multifunctional microplate reader (MB-530, HEALES, China).

### 2.14. Flow Cytometry

The digestion of skeletal myoblasts was accomplished using EDTA-free trypsin. After rinsing with PBS, about 3.2 × 10^5^ cells were collected. The cell mixture was combined with 5 μL of Annexin V-FITC and 5 μL of propidium iodide in compliance with the guidelines of the apoptosis kit (KGA108, KeyGEN BioTECH, China). After a 10-min lightless incubation, an assessment of the cells was conducted utilizing a flow cytometer (A00-1-1102, Beckman, USA).

### 2.15. Statistical Analysis

The analysis of experimental data was executed using GraphPad Prism 8.0 (GraphPad Software Inc., USA) and expressed as mean ± standard deviation of three independent experiments. The measurement data adhered to the normal distribution and homogeneity of variance, which were analyzed by the Kolmogorov–Smirnov test and exploratory descriptive statistics test. A one-way analysis of variance (ANOVA) along with Tukey's post hoc test was employed for the comparison of multiple groups. *p* < 0.05 was considered statistically significant. All investigations were carried out in a randomized and blind way to reduce any likelihood of experimental bias.

## 3. Results

### 3.1. THSWT Improved LIRI in Rats

**Figure 1:**
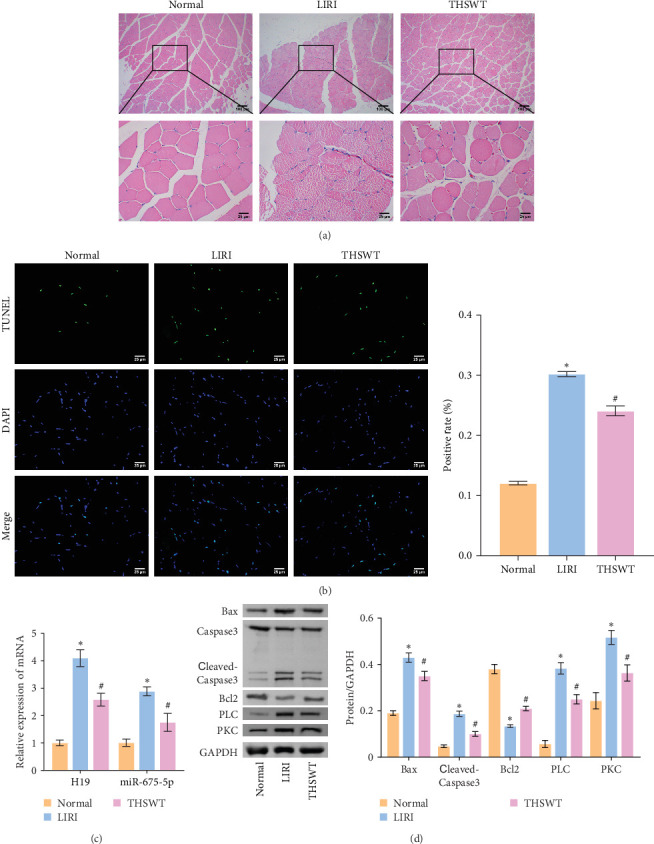
Taohong Siwu decoction (THSWT) had the potential to improve limb ischemia/reperfusion injury (LIRI) in rats. (A) The pathological changes in skeletal muscle were observed by H&E staining. (B) Cell apoptosis in skeletal muscle was assessed by TUNEL staining. (C) The expressions of H19 and miR-675-5p in skeletal muscle were evaluated by quantitative real-time polymerase chain reaction (qRT-PCR). (D) The expressions of cleaved-Caspase3, Bax, Bcl2, PLC, and PKC were assessed by western blot. *⁣*^*∗*^*p* < 0.05 vs. Normal; ^#^*p* < 0.05 vs. LIRI.

To explore the impact of THSWT on LIRI, SD rat models of LIRI were constructed using an improved method of blocking blood flow in the femoral artery on the body surface. These model rats were then subjected to intragastric THSWT intervention. H&E staining displayed that skeletal muscle cells in the Normal group exhibited normal morphology, with intact cell membranes and an orderly arrangement of muscle fibers. In contrast, the LIRI group showed disorder with damaged integrity and continuity in some areas. However, in the THSWT group, there was a remarkable improvement in skeletal muscle injury, with basically intact cell structures and relatively neat muscle fiber arrangement (Figure 1A). Further analysis demonstrated that the cell apoptosis rate in skeletal muscle was raised in the LIRI group compared with normal rats. However, THSWT intervention effectively reduced LIRI-induced cell apoptosis in skeletal muscle (Figure 1B). Additionally, the THSWT intervention downregulated LIRI-induced high expressions of H19 and miR-675-5p (Figure 1C). Western blot results displayed that LIRI induced upregulated expressions of cleaved-Caspase3, Bax, PLC, and PKC, while downregulated expression of Bcl2. However, the THSWT intervention reversed these altered protein expressions induced by LIRI (Figure 1D). Overall, these results suggested that THSWT could improve LIRI in rats.

### 3.2. Silencing of H19 Downregulated miR-675-5p Expression and Improved LIRI in Rats

**Figure 2:**
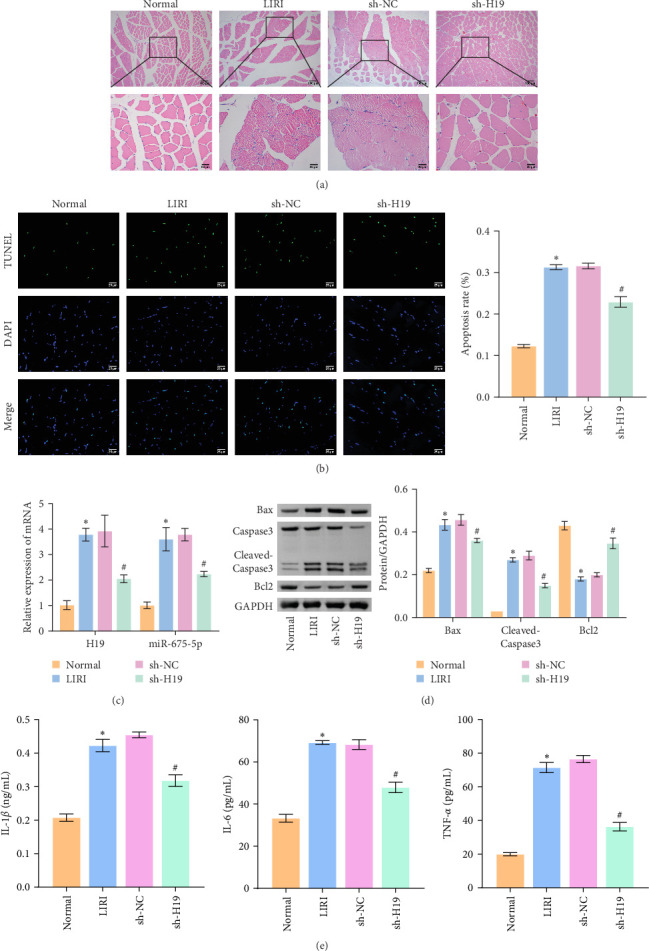
Silencing of H19 could inhibit miR-675-5p to improve limb ischemia/reperfusion injury (LIRI) in rats. (A) The pathological changes in skeletal muscle were observed by H&E staining. (B) Cell apoptosis in skeletal muscle was assessed by TUNEL staining. (C) The expressions of H19 and miR-675-5p in skeletal muscle were evaluated by quantitative real-time polymerase chain reaction (qRT-PCR). (D) The expressions of cleaved-Caspase3, Bax, and Bcl2 were analyzed by western blot. (E) Serum levels of IL-1*β*, IL-6, and TNF-*α* were assessed by enzyme-linked immunosorbent assay (ELISA). *⁣*^*∗*^*p* < 0.05 vs. Normal; ^#^*p* < 0.05 vs. LIRI; ^&^*p* < 0.05 vs. sh-NC.

To clarify the regulatory role of the H19/miR-675-5p axis in the occurrence and development of LIRI, H19 was silenced in LIRI rats. The results displayed that silencing of H19 improved the destruction of skeletal muscle cells induced by LIRI, along with relatively complete cell structure and relatively well-aligned muscle fibers (Figure 2A). Further, silencing of H19 reduced LIRI-induced cell apoptosis in skeletal muscle (Figure 2B). Notably, the silencing of H19 downregulated LIRI-induced high expressions of H19 and miR-675-5p (Figure 2C). Similarly, the high expressions of cleaved-Caspase3 and Bax induced by LIRI were downregulated after silencing of H19, while the expression of Bcl2 was the opposite (Figure 2D). Analysis of serum inflammatory cytokine levels showed an increase in IL-1*β*, IL-6, and TNF-*α* levels after LIRI. After the silencing of H19, the release of these inflammatory cytokines was inhibited (Figure 2E). Taken together, these results illustrated that silencing of H19 could downregulate miR-675-5p expression and improve LIRI in rats.

### 3.3. Combination of Silencing of miR-675-5p and THSWT Intervention Improved LIRI in Rats

**Figure 3:**
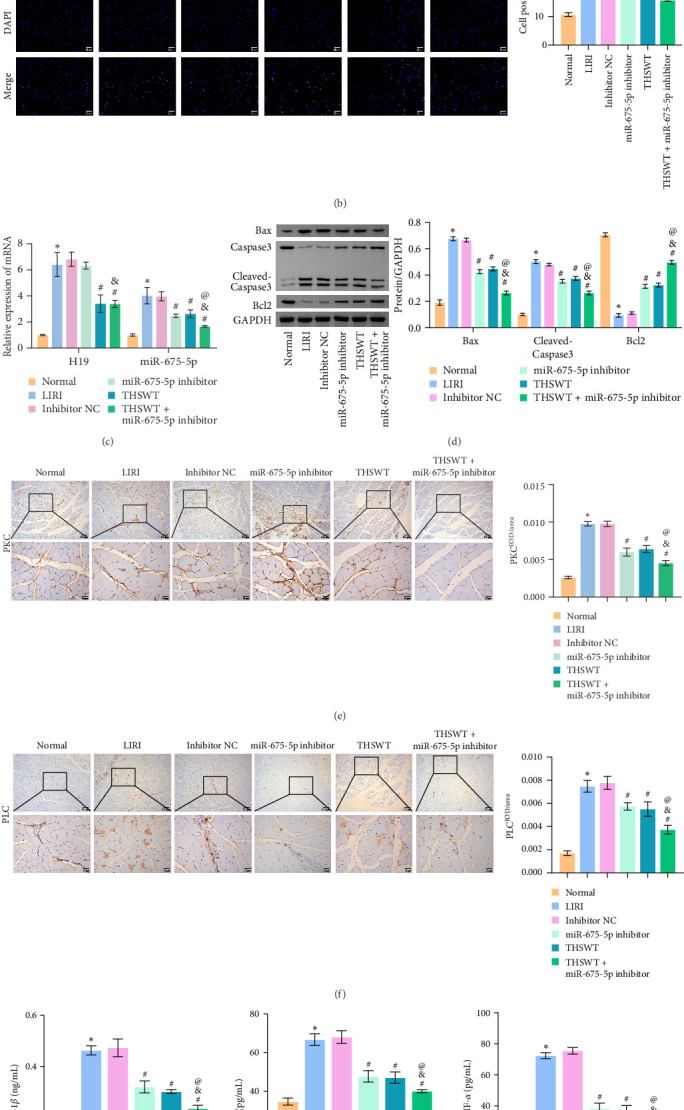
Taohong Siwu decoction (THSWT) intervention combined with silencing of miR-675-5p could improve limb ischemia/reperfusion injury (LIRI) in rats. (A) The pathological changes in skeletal muscle were observed by H&E staining. (B) Cell apoptosis in skeletal muscle was assessed by TUNEL staining. (C) The expressions of H19 and miR-675-5p in skeletal muscle were evaluated by quantitative real-time polymerase chain reaction (qRT-PCR). (D) The expressions of cleaved-Caspase3, Bax, and Bcl2 were analyzed by Western blot. (E, F) The expressions of PLC and PKC in skeletal muscle were analyzed by immunohistochemistry (IHC) staining. (G) Serum levels of IL-1*β*, IL-6, and TNF-*α* were assessed by enzyme-linked immunosorbent assay (ELISA). *⁣*^*∗*^*p* < 0.05 vs. Normal; ^#^*p* < 0.05 vs. LIRI; ^&^*p* < 0.05 vs. miR-675-5p inhibitor; ^@^*p* < 0.05 vs. THSWT.

In the following study, we investigated the impact of the combination of silencing of miR-675-5p and THSWT intervention on LIRI in rats. We observed that silencing of miR-675-5p and THSWT intervention reduced, respectively, the injury of skeletal muscle cells induced by LIRI. However, the combined intervention greatly saved skeletal muscle cell damage in LIRI rats (Figure 3A). Additionally, both silencing of miR-675-5p and THSWT intervention inhibited LIRI-induced apoptosis occurrence of skeletal muscle cells. The combined intervention showed stronger inhibition of apoptosis of skeletal muscle cells (Figure 3B). In the skeletal muscle of LIRI rats, H19 expression was not affected by silencing of miR-675-5p, but downregulated after THSWT intervention. Furthermore, miR-675-5p expression was both downregulated by silencing of miR-675-5p and THSWT intervention, and downregulated more under the combined intervention (Figure 3C). Moreover, the expressions of cleaved-Caspase3, Bax, PLC, and PKC were inhibited, while the expression of Bcl2 was the opposite after silencing of miR-675-5p or THSWT intervention in LIRI rats. The combined intervention regulated these proteins more strongly than either one (Figure 3D–F). Additionally, silencing of miR-675-5p and THSWT intervention reduced the inflammatory response in LIRI rats, which was represented by reduced serum levels of IL-1*β*, IL-6, and TNF-*α*. As expected, the combined intervention further reduced the inflammatory response in LIRI rats (Figure 3G). These findings proved that the combination of silencing of miR-675-5p and THSWT intervention could improve LIRI in rats.

### 3.4. THSWT Combined With H19/miR-675-5p Axis Regulated the Wnt3 a/Ca^2+^ Signaling Pathway

**Figure 4:**
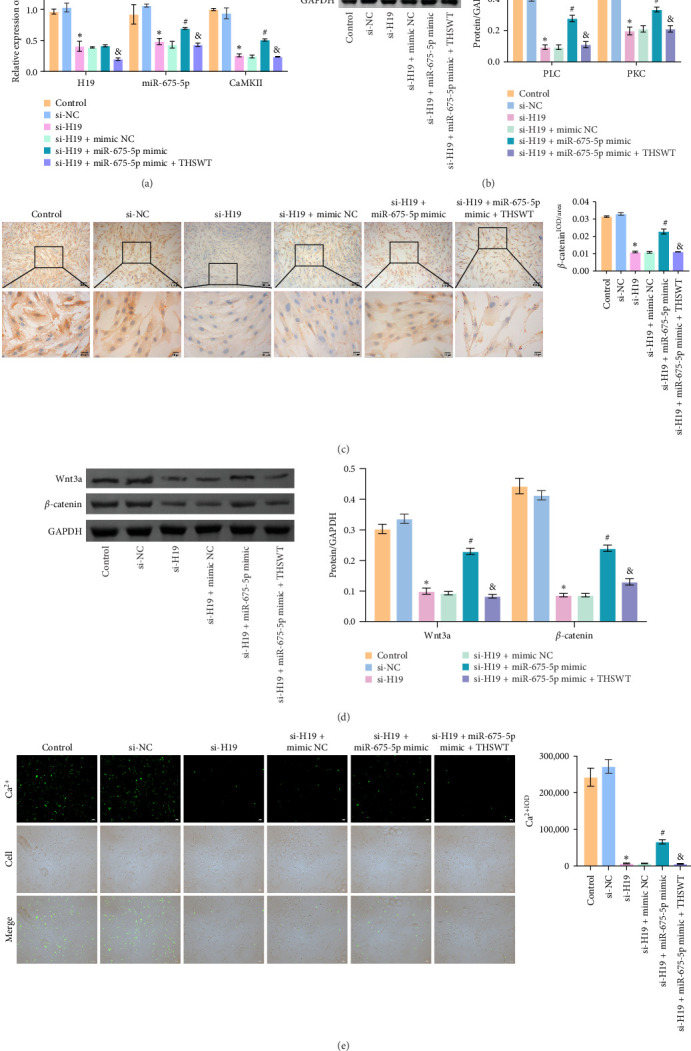
Taohong Siwu decoction (THSWT) combined with the H19/miR-675-5p axis could regulate the wingless/integrated 3a (Wnt3a)/Ca^2+^ signaling pathway. (A) The expressions of H19, miR-675-5p, and CaMKⅡ in skeletal myoblasts were evaluated by quantitative real-time polymerase chain reaction (qRT-PCR). (B) The expressions of PLC and PKC in skeletal myoblasts were analyzed by western blot. (C) The expression of *β*-catenin in skeletal myoblasts was analyzed by immunohistochemistry (IHC) staining. (D) The expressions of Wnt3a and *β*-catenin in skeletal myoblasts were analyzed by Western blot. (E) Determination of Ca^2+^ concentration in skeletal myoblasts by calcium fluorescence imaging. *⁣*^*∗*^*p* < 0.05 vs. si-NC; ^#^*p* < 0.05 vs. si-H19+mimic NC; ^&^*p* < 0.05 vs. si-H19+miR-675-5p mimic.

To elucidate the potential mechanism of THSWT combined with the H19/miR-675-5p axis in LIRI treatment, we constructed a cell model by treating skeletal myoblasts with a simulated IR solution. Cells were subjected to H19 silencing, miR-675-5p overexpression, and treatment with a THSWT-containing medium or a combination of the above methods for intervention. The successful and efficient H19 silencing and miR-675-5p overexpression were verified by qRT-PCR. Silencing of H19 decreased the expressions of miR-675-5p and CaMKⅡ in injured skeletal myoblasts. However, overexpression of miR-675-5p partially offset the impact of silencing of H19 on the above indicators. However, further treatment with THSWT-containing medium reduced the expressions of H19, miR-675-5p, and CaMKⅡ in injured skeletal myoblasts (Figure 4A). Western blot results displayed that overexpression of miR-675-5p inhibited the downregulation of PLC and PKC induced by silencing of H19. However, further treatment with a THSWT-containing medium counteracted the harmful effects of overexpression of miR-675-5p, showing reduced expressions of PLC and PKC (Figure 4B). Further, level of *β*-catenin decreased after silencing of H19. However, overexpression of miR-675-5p reversed these changes. When injured skeletal myoblasts were further treated with THSWT-containing medium, level of *β*-catenin was greatly reduced (Figure 4C). Silencing of H19 inhibited the Wnt3a and *β*-catenin proteins in skeletal myoblasts with a simulated IR solution. Overexpression of miR-675-5p inhibited the downregulation of Wnt3a and *β*-catenin proteins induced by silencing of H19. However, further treatment with a THSWT-containing medium counteracted the harmful effects of overexpression of miR-675-5p, showing reduced expressions of Wnt3a and *β*-catenin proteins (Figure 4D). Moreover, level of Ca^2+^ decreased after silencing of H19. However, overexpression of miR-675-5p reversed these changes. When injured skeletal myoblasts were further treated with THSWT-containing medium, level of Ca^2+^ were greatly reduced (Figure 4E). These results illustrated that THSWT combined with the H19/miR-675-5p axis could regulate the Wnt3a/Ca^2+^ signaling pathway.

### 3.5. THSWT Intervention Combined with the H19/miR-675-5p Axis Regulation Repaired LIRI

**Figure 5:**
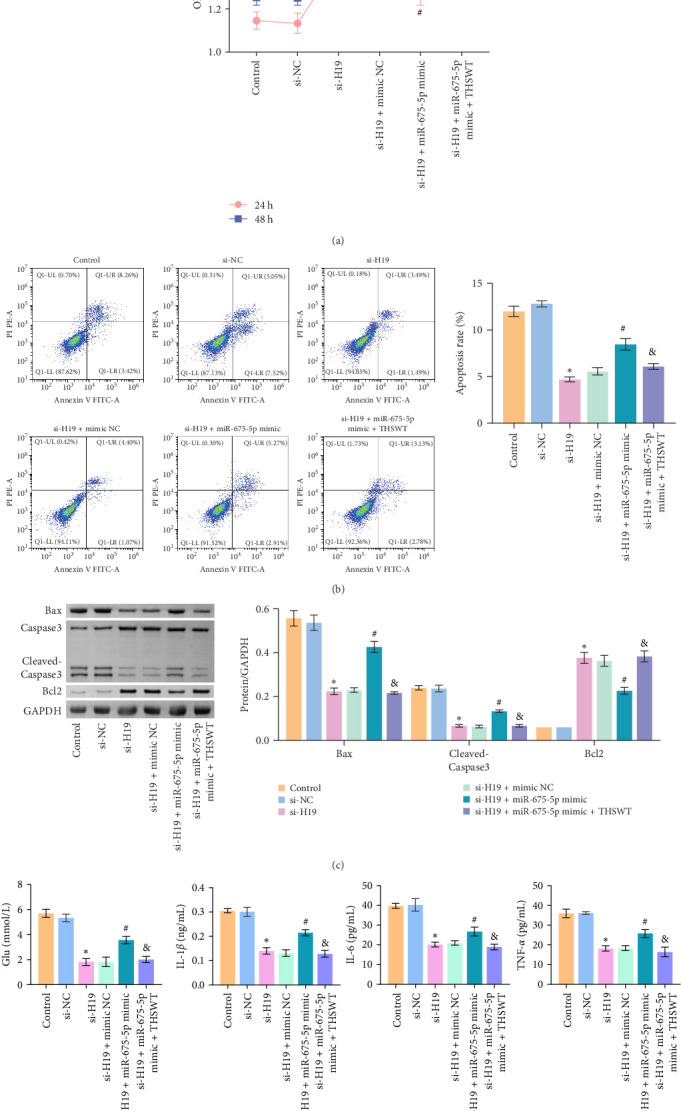
The combination of Taohong Siwu decoction (THSWT) intervention and the H19/miR-675-5p axis regulation repaired limb ischemia/reperfusion injury (LIRI). (A) The proliferation of skeletal myoblasts was evaluated using the cell counting kit-8 (CCK-8) assay. (B) Cell apoptosis of skeletal myoblasts was analyzed using flow cytometry. (C) Expressions of cleaved-Caspase3, Bax, and Bcl2 in skeletal myoblasts were analyzed by western blot. (D) The glucose (Glu) level in the supernatant of skeletal myoblasts was measured using a biochemical assay kit. (E) Levels of IL-1*β*, IL-6, and TNF-*α* in the supernatant of skeletal myoblasts were evaluated by enzyme-linked immunosorbent assay (ELISA). *⁣*^*∗*^*p* < 0.05 vs. si-NC; ^#^*p* < 0.05 vs. si-H19+mimic NC; ^&^*p* < 0.05 vs. si-H19+miR-675-5p mimic.

According to the results mentioned above, we further confirmed the efficacy of THSWT intervention combined with the H19/miR-675-5p axis regulation in repairing LIRI by modulating the Wnt/Ca^2+^ signaling pathway. The results showed that overexpression of miR-675-5p partially counteracted the promotion of silencing of H19 on the proliferation of injured skeletal myoblasts. However, further treatment with THSWT-containing medium conversed the trends above, resulting in a restored proliferation of injured skeletal myoblasts (Figure 5A). Moreover, overexpression of miR-675-5p changed the apoptosis inhibition of silencing of H19 on injured skeletal myoblasts, exhibiting increased expressions of cleaved-Caspase3 and Bax and decreased expression of Bcl2. However, further treatment with THSWT-containing medium counteracted the harmful effects of overexpression of miR-675-5p, showing decreased expressions of cleaved-Caspase3 and Bax and increased expression of Bcl2 (Figure 5B,C). Furthermore, the levels of Glu, IL-1*β*, IL-6, and TNF-*α* in the cell culture supernatant decreased under the influence of silencing of H19. However, overexpression of miR-675-5p reversed the effect of silencing of H19. When further treated with THSWT-containing medium, the levels of Glu, IL-1*β*, IL-6, and TNF-*α* in the cell culture supernatant were reduced (Figure 5D,E). These results displayed that THSWT intervention combined with the H19/miR-675-5p axis regulation could repair LIRI.

## 4. Discussion

Among orthopedic diseases, LIRI has high disability and mortality rates [2]. Currently, the main treatment options for LIRI in clinical practice include fasciotomy, hyperbaric oxygen therapy, gene therapy, et cetera. However, these treatments are costly, have unstable efficacy, and their mechanisms of action are not fully understood [35–37]. With the development of TCM, many Chinese herbs and their active ingredients have shown advantages in the treatment of various types of IRI [7, 8, 38]. Currently, most studies believe that LIRI is associated with calcium metabolism disorder, cell apoptosis, and inflammatory damage. At the molecular pathological level, targeted regulation of signaling molecules has gradually become a research hotspot [2, 39–41]. In this study, the targeted regulation of the THSWT combined with the H19/miR-675-5p axis effectively repaired LIRI. Mechanistically, this therapeutic effect was achieved through the regulation of the Wnt3a/Ca^2+^ signaling pathway.

IRI exhibits multiple pathological changes, with cell apoptosis and inflammation being two core aspects. Inflammatory cytokines like TNF-*α*, IL-1*β*, and IL-6 participate in the pathological physiology of IRI. Among them, TNF-*α* is the initiating factor of the inflammatory cascade reaction and has been proven to be one of the therapeutic targets for various types of IRI [42]. Studies have shown that the administration of anti-TNF-*α* antibodies at the beginning of reperfusion can alleviate ischemic injury [43]. For example, the TCM ingredient Naoyanghuasu III inhibits the TNF-*α*-induced angiogenesis and inflammatory response of human umbilical vein endothelial cells by regulating the NIK/IKK-*α*/CXCL12 pathway [44]. TNF-*α* induces monocyte macrophages to release IL-1*β* and IL-6, showing a synergistic pro-inflammatory effect. Downregulating IL-1*β* expression has been shown to protect the lungs of intestinal IRI rats from acute injury [45]. In the study of myocardial IRI, IL-6 has been found to contribute to the development of early reperfusion myocardial infarction [46]. In this study, TNF-*α*, IL-1*β*, and IL-6 levels in the serum of LIRI rats were raised compared to the Normal group. This also confirmed the essentially inflammatory nature of the pathological process in LIRI. However, the release of TNF-*α*, IL-1*β*, and IL-6 was inhibited after THSWT intervention, which may be due to the anti-inflammatory activity of the active ingredients in THSWT. For example, the neuroprotective effect of safflower flavonoid extract may be related to its anti-inflammatory effect [47]. In addition, liguzinediol can inhibit the synthesis of pro-inflammatory factors to alleviate congestive heart failure caused by myocardial infarction [48]. In the pathological process of IRI, cell apoptosis and inflammation are interdependent. Cell apoptosis releases a large number of intracellular substances into the surrounding tissue gaps, exacerbating the infiltration of inflammatory cells and inflammatory reactions [49, 50]. Studies have shown that garlic can inhibit cell apoptosis and alleviate skeletal muscle injury induced by IR by reducing the expression of IL-1*β* and caspase-3 [51]. Moreover, the activation of the PKC/NADPH oxidase pathway can promote IR-induced cell apoptosis [52]. The polysaccharides from *A. sinensis* in THSWT can alleviate neuronal apoptosis induced by focal cerebral ischemia [53]. In this study, THSWT suppressed LIRI-induced cell apoptosis, manifested as downregulation of cleaved-Caspase3 and Bax expression, and upregulation of Bcl2 expression in the skeletal muscle. These studies confirm that THSWT inhibits IR induced apoptosis injury, which will contribute to its further application.

H19 is a 3.0 kb LncRNA with highly conserved evolution. It involves multiple types of IRI and can be considered a potential target for IRI treatment. In cerebral IRI, the expression of H19 is upregulated under ischemic conditions, promoting cell death through autophagy and exacerbating brain damage [54]. In addition, H19 promotes neuronal necroptosis through the miR-181a-5p/HMGB1 axis, leading to SCIRI [21]. Studies have shown that H19 is a precursor of miR-675. The H19/miR-675 axis participates in the occurrence and metastasis of cancers [55, 56]. However, the regulatory mechanism of the H19/miR-675 axis in LIRI has not been reported. Here, the expressions of both H19 and miR-675-5p were upregulated after LIRI. Silencing of H19 and inhibiting miR-675-5p improved the damage to skeletal muscle caused by LIRI. When combined with THSWT intervention, the reparative effect on LIRI was further enhanced. These results illustrate that THSWT may improve LIRI through the inhibition of the H19/miR-675-5p axis in skeletal muscle.

Studies have shown that a key process in IRI is intracellular calcium overload in tissues [57]. To further clarify the therapeutic mechanism of THSWT in LIRI, the expressions of proteins related to calcium homeostasis and the Wnt/Ca^2+^ pathway were analyzed. Most studies have shown that the Wnt/Ca^2+^ signaling pathway is involved in embryonic development, tissue formation, and diseases [58]. However, there are few studies on the expression of the Wnt/Ca^2+^ signaling pathway and related proteins in LIRI. It has been shown that Wnt-*β*-catenin signal transduction can protect mice from liver IRI [59]. CaMKⅡ plays a crucial role in calcium transfer and can combine with calcium to form a Ca^2+^/CaM complex [60]. In the IR process, excessive calcium enters the cells, activating the Ca^2+^/CaM complex. In this study, LIRI induced intracellular calcium overload. THSWT intervention combined with the H19/miR-675-5p axis regulation reduced the levels of PLC, PKC, CaMKⅡ, Wnt3a, *β*-catenin, and Ca^2+^. These results are consistent with our previous findings [34]. These results suggest that the Wnt/Ca^2+^ pathway may be a regulatory target of THSWT in LIRI.

DADLE improves left ventricular function in a mouse IR model by inhibiting the expression of Wnt3a and *β*-Catenin, exerting a protective effect against myocardial IRI [61]. During the differentiation of human neuronal progenitor cells, Wnt3a triggers the Wnt/Ca^2+^ signaling pathway, leading to an increase in cytosolic free calcium ([Ca^2+^]i) and activation of calcium/calmodulin-dependent kinase II [62]. Wnt3a rapidly induces O-glycosylation through the Ca^2+^-PKA-Gfat1 axis or increases O-glycosylation in a Wnt-*β*-catenin-dependent manner after prolonged stimulation, stabilizing PDK1 protein and enhancing glycolysis and osteogenic activity [63]. In this study, we found that THSWT alone or in combination with LncRNA H19/miR-675-5p axis regulates Wnt3a/*β*-catenin and Ca^2+^, consistent with the above findings. Our study simulated IR solution for simulating LIRI environment and preliminarily confirmed the mechanism of action of THSWT. Although this modeling method has been widely used, the lack of low oxygen group treatment remains one of the limitations of this study. Other studies have reported that Wnt-5a, an atypical Wnt ligand, regulates neuronal development and differentiation by modulating the CDK5 pathway through Ca^2+^/calpain signaling [64]. After simulating IR solution treatment, the secretion of Wnt5a/Ca^2+^ signaling molecules and intracellular Ca^2+^ concentration in myoblasts increased, but were weakened by THSWT treatment [34]. These study may encourage the use of THSWT in critical clinical settings for LIRI.

Despite the promising results and potential therapeutic benefits of using THSWT and H19/miR-675-5p axis in the treatment of LIRI, several limitations should be considered. First, the study focuses on animal and cell models, and there may be differences in the response and treatment outcomes compared to human patients. It is crucial to conduct further research and clinical trials to validate the findings in human subjects and assess the safety and efficacy of these treatments. Additionally, the study primarily examined the effects of THSWT and the H19/miR-675-5p axis on specific targets within the Wnt3a/Ca^2+^ signaling pathway. However, the interaction and crosstalk between different pathways and the potential synergistic or antagonistic effects of multiple targets should be considered. A comprehensive understanding of the complex molecular interactions involved in LIRI will enhance the development of more effective treatments.

## 5. Conclusion

To summarize, our study demonstrated that the combination of THSWT intervention with the regulation of the H19/miR-675-5p axis effectively improved LIRI by modulating the Wnt3a/Ca^2+^ signaling pathway. These findings provided valuable insights into potential therapeutic strategies for LIRI. The next steps could focus on further investigating the detailed mechanisms of the H19/miR-675-5p axis and exploring potential clinical applications of THSWT in LIRI.

## Figures and Tables

**Table 1:** Primer sequences.

Gene	Sequences		Length (bp)
	Forward (5′−3′)	Reverse (5′−3′)	
R-GAPDH	ACAGCAACAGGGTGGTGGAC	TTTGAGGGTGCAGCGAACTT	252
R-H19	CTGTATGCCCTAACCGCTCAGT	CTCTACCTGCACCCAACCTCC	151
R-miR-675-5p	TGGTGCGGAAAGGGCCCACAGT	GCTGTCAACGATACGCTACGTAA	77
R-CAMK2a	GAGCACCAACACCACCATCGAG	CGGTTCAAAGGCTGTCATTCCA	151

**Table 2:** The information about antibodies.

Name	Dilution rate	Cat number	Source	Company	Country
Bax	1:5000	ab32503	Rabbit	Abcam	UK
Caspase-3	1:1000	19677-1-AP	Rabbit	Proteintech	USA
Bcl2	1:2000	ab182858	Rabbit	Abcam	UK
PLC	1:1000	ab41433	Mouse	Abcam	UK
PKC	1:5000	ab32376	Rabbit	Abcam	UK
Wnt3a	1:1000	26744-1-AP	Rabbit	Proteintech	USA
*β*-catenin	1:20000	51067-2-AP	Rabbit	Proteintech	USA
GAPDH	1:5000	10494-1-AP	Rabbit	Proteintech	USA
HRP goat anti-mouse IgG	1:5000	SA00001-1	Mouse	Proteintech	USA
HRP goat anti-Rabbit IgG	1:6000	SA00001-2	Rabbit	Proteintech	USA

Abbreviation: Wnt, wingless/integrated.

## Data Availability

The data that support the findings of this study are available from the corresponding author upon reasonable request.

## References

[1] Sun H., Wang J., Bi W. (2022). Dl-3-n-Butylphthalide (NBP) Mitigates Muscular Injury Induced by Limb Ischemia/Reperfusion in Mice Through the HMGB1/TLR4/NF-*κ*B Pathway. *Evidence-Based Complementary and Alternative Medicine*.

[2] Foster A. D., Vicente D., Sexton J. J. (2017). Administration of FTY720 During Tourniquet-Induced Limb Ischemia Reperfusion Injury Attenuates Systemic Inflammation. *Mediators of Inflammation*.

[3] Blaisdell F. W. (2002). The Pathophysiology of Skeletal Muscle Ischemia and the Reperfusion Syndrome: A Review. *Cardiovascular Surgery*.

[4] Gillani S., Cao J., Suzuki T., Hak D. J. (2012). The Effect of Ischemia Reperfusion Injury on Skeletal Muscle. *Injury*.

[5] Harkin D. W., Barros D’Sa A. A. B., Yassin M. M. I. (2001). Recombinant Bactericidal/Permeability-Increasing Protein Attenuates the Systemic Inflammatory Response Syndrome in Lower Limb Ischemia-Reperfusion Injury. *Journal of Vascular Surgery*.

[6] Homer-Vanniasinkam S., Crinnion J. N., Gough M. J. (1997). Post-Ischaemic Organ Dysfunction: A Review. *European Journal of Vascular and Endovascular Surgery*.

[7] Wang R., Wang M., Zhou J. (2021). Saponins in Chinese Herbal Medicine Exerts Protection in Myocardial Ischemia–Reperfusion Injury: Possible Mechanism and Target Analysis. *Frontiers in Pharmacology*.

[8] Xing N., Long X. T., Zhang H. J. (2022). Research Progress on Effects of Traditional Chinese Medicine on Myocardial Ischemia-Reperfusion Injury: A Review. *Frontiers in Pharmacology*.

[9] Yu Y. W., Liu S., Zhou Y. Y. (2022). Shexiang Baoxin Pill Attenuates Myocardial Ischemia/Reperfusion Injury by Activating Autophagy via Modulating the ceRNA-Map3k8 Pathway. *Phytomedicine*.

[10] Peng T., Jiang Y., Farhan M., Lazarovici P., Chen L., Zheng W. (2019). Anti-Inflammatory Effects of Traditional Chinese Medicines on Preclinical in Vivo Models of Brain Ischemia-Reperfusion-Injury: Prospects for Neuroprotective Drug Discovery and Therapy. *Frontiers in Pharmacology*.

[11] Liu T. H., Chen W. H., Chen X. D. (2020). Network Pharmacology Identifies the Mechanisms of Action of TaohongSiwu Decoction Against Essential Hypertension. *Medical Science Monitor*.

[12] Shuang-Shuang L. I., Chun-Yan G., D. O. Pharmacy, H. N. University (2016). Advances on Chemical Constituents and Pharmacological Effects of Taohongsiwu Decoction. *Acta Neuropharmacologica*.

[13] Zhang X., Li P., Hua Y. (2018). Urinary Metabolomics Study the Mechanism of Taohong Siwu Decoction Intervention in Acute Blood Stasis Model Rats Based on Liquid Chromatography Coupled to Quadrupole Time-of-Flight Mass Spectrometry. *Journal of Chromatography B*.

[14] Li L., Yang N., Nin L. (2015). Chinese Herbal Medicine Formula Tao Hong Si Wu Decoction Protects Against Cerebral Ischemia-Reperfusion Injury via PI3K/Akt and the Nrf2 Signaling Pathway. *Journal of Natural Medicines*.

[15] Lu X., Li J., Zhou B., Lu X., Li W., Ouyang J. (2023). Taohong Siwu Decoction Enhances Human Bone Marrow Mesenchymal Stem Cells Proliferation, Migration and Osteogenic Differentiation via VEGF-FAK Signaling in Vitro. *Journal of Ethnopharmacology*.

[16] Tang Z., Yin M., Guo Y. (2022). Taohong Siwu Decoction Promotes Osteo-Angiogenesis in Fractures by Regulating the HIF-1*α* Signaling Pathway. *Evidence-Based Complementary and Alternative Medicine*.

[17] Xu Y. L., Zhang M. H., Guo W. (2018). MicroRNA-19 Restores Vascular Endothelial Cell Function in Lower Limb Ischemia-Reperfusion Injury Through the KLF10-Dependent TGF-*β*1/Smad Signaling Pathway in Rats. *Journal of Cellular Biochemistry*.

[18] Zhang S., Zhu T., Li Q., Sun G., Sun X. (2021). Long Non-Coding RNA-Mediated Competing Endogenous RNA Networks in Ischemic Stroke: Molecular Mechanisms, Therapeutic Implications, and Challenges. *Frontiers in Pharmacology*.

[19] Cao M., Song W., Liang R. (2021). MicroRNA as a Potential Biomarker and Treatment Strategy for Ischemia-Reperfusion Injury. *International Journal of Genomics*.

[20] Luo H., Wang J., Liu D. (2019). The lncRNA H19/miR-675 Axis Regulates Myocardial Ischemic and Reperfusion Injury by Targeting PPAR*α*. *Molecular Immunology*.

[21] Guo L., Wang D., Alexander H. Y., Ren X., Ma H. (2022). Long Non-Coding RNA H19 Contributes to Spinal Cord Ischemia/Reperfusion Injury Through Increasing Neuronal Pyroptosis by miR-181a-5p/HMGB1 Axis. *Aging (Albany NY)*.

[22] Costa V., Raimondi L., Conigliaro A. (2017). Hypoxia-Inducible Factor 1*A* May Regulate the Commitment of Mesenchymal Stromal Cells Toward Angio-Osteogenesis by mirna-675-5P. *Cytotherapy*.

[23] Regan J. N., Waning D. L., Guise T. A. (2016). Skeletal Muscle Ca^2+^ Mishandling: Another Effect of Bone-to-Muscle Signaling. *Seminars in Cell & Developmental Biology*.

[24] Bellinger A. M., Reiken S., Carlson C. (2009). Hypernitrosylated Ryanodine Receptor Calcium Release Channels Are Leaky in Dystrophic Muscle. *Nature Medicine*.

[25] Andersson D. C., Betzenhauser M. J., Reiken S. (2011). Ryanodine Receptor Oxidation Causes Intracellular Calcium Leak and Muscle Weakness in Aging. *Cell Metabolism*.

[26] Huybrechts Y., Mortier G., Boudin E., Van Hul W. (2020). WNT Signaling and Bone: Lessons From Skeletal Dysplasias and Disorders. *Frontiers in Endocrinology*.

[27] Flores-Hernández E., Velázquez D. M., Castañeda-Patlán M. C. (2020). Canonical and Non-Canonical Wnt Signaling Are Simultaneously Activated by Wnts in Colon Cancer Cells. *Cellular Signalling*.

[28] Sarabia-Sánchez M. A., Moreno-Londoño A. P., Castañeda-Patlán M. C., Alvarado-Ortiz E., Martínez-Morales J. C., Robles-Flores M. (2023). Non-Canonical Wnt/Ca^2+^ Signaling Is Essential to Promote Self-Renewal and Proliferation in Colon Cancer Stem Cells. *Frontiers in Oncology*.

[29] Zhang G., Ding L., Sun G. LncRNA AZIN1-AS1 Ameliorates Myocardial Ischemia-Reperfusion Injury by Targeting miR-6838-5p/WNT3A Axis to Activate Wnt-β/Catenin Signaling Pathway. *In Vitro Cellular & Developmental Biology. Animal*.

[30] Zhang G., Ge M., Han Z. (2019). Wnt/*β*-Catenin Signaling Pathway Contributes to Isoflurane Postconditioning Against Cerebral Ischemia-Reperfusion Injury and Is Possibly Related to the Transforming Growth Factor*β*1/Smad3 Signaling Pathway. *Biomedicine & Pharmacotherapy*.

[31] Chen X., Yao N., Mao Y. (2024). Activation of the Wnt/*β*-Catenin/CYP1B1 Pathway Alleviates Oxidative Stress and Protects the Blood-Brain Barrier Under Cerebral Ischemia/Reperfusion Conditions. *Neural Regeneration Research*.

[32] Salvaterra C. G., Goldman W. F. (1993). Acute Hypoxia Increases Cytosolic Calcium in Cultured Pulmonary Arterial Myocytes. *American Journal of Physiology-Lung Cellular and Molecular Physiology*.

[33] Koyama T., Temma K., Akera T. (1991). Reperfusion-Induced Contracture Develops With a Decreasing [Ca^2+^]i in Single Heart Cells. *American Journal of Physiology-Heart and Circulatory Physiology*.

[34] Fuping Z., Wuping L., Linhua W. (2019). Tao-Hong-Si-Wu Decoction Reduces Ischemia Reperfusion Rat Myoblast Cells Calcium Overloading and Inflammation Through the Wnt/IP3R/CAMKII Pathway. *Journal of Cellular Biochemistry*.

[35] Tsuchihara T., Nukada H., Nakanishi K. (2020). Efficacy of Nonviral Gene Transfer of Human Hepatocyte Growth Factor (HGF) Against Ischemic-Reperfusion Nerve Injury in Rats. *PLoS One*.

[36] Frisby D., Tu H., Qian J. (2021). Hyperbaric Oxygen Therapy Does Not Alleviate Tourniquet-Induced Acute Ischemia-Reperfusion Injury in Mouse Skeletal Muscles. *Injury*.

[37] Orrapin S., Orrapin S., Arwon S., Rerkasem K. (2017). Predictive Factors for Post-Ischemic Compartment Syndrome in Non-Traumatic Acute Limb Ischemia in a Lower Extremity. *Annals of Vascular Diseases*.

[38] Yuan Q., Yuan Y., Zheng Y. (2021). Anti-Cerebral Ischemia Reperfusion Injury of Polysaccharides: A Review of the Mechanisms. *Biomedicine & Pharmacotherapy*.

[39] Aby K., Antony R., Li Y. (2023). ProBDNF Upregulation in Murine Hind Limb Ischemia Reperfusion Injury: A Driver of Inflammation. *Biology (Basel)*.

[40] Joshi D., Abraham D., Shiwen X., Baker D., Tsui J. (2014). Potential Role of Erythropoietin Receptors and Ligands in Attenuating Apoptosis and Inflammation in Critical Limb Ischemia. *Journal of Vascular Surgery*.

[41] Tong Z., Yu F., Liu Z., Liang H. (2012). Influence of ShuJinHuoXue Tablets on Ischemia Reperfusion Injury of Animals’ Skeletal Muscle. *Molecules*.

[42] Esposito E., Cuzzocrea S. (2009). TNF-Alpha as a Therapeutic Target in Inflammatory Diseases, Ischemia- Reperfusion Injury and Trauma. *Current Medicinal Chemistry*.

[43] Li W., Liu D., Xu J. (2022). Astrocyte-Derived TNF-*α*-Activated Platelets Promote Cerebral Ischemia/Reperfusion Injury by Regulating the RIP1/RIP3/AKT Signaling Pathway. *Molecular Neurobiology*.

[44] Liu X., Li S., Xu Y., Mei W., Zhou R. (2023). A Study on the Pharmacological Effects and Mechanism of Rhodojaponin III in Rheumatoid Arthritis. *Frontiers in Bioscience-Landmark*.

[45] Zheng D. Y., Zhou M., Jin J. (2016). Inhibition of P38 MAPK Downregulates the Expression of IL-1 *β* to Protect Lung From Acute Injury in Intestinal Ischemia Reperfusion Rats. *Mediators of Inflammation*.

[46] Jong W. M., Cate H. T., Linnenbank A. C. (2016). Reduced Acute Myocardial Ischemia-Reperfusion Injury in IL-6-Deficient Mice Employing a Closed-Chest Model. *Inflammation Research*.

[47] Lei H., Ren R., Sun Y. (2020). Neuroprotective Effects of Safflower Flavonoid Extract in 6-Hydroxydopamine-Induced Model of Parkinson’s Disease May be Related to Its Anti-Inflammatory Action. *Molecules*.

[48] Chen Q., Zhang D., Bi Y. (2020). The Protective Effects of Liguzinediol on Congestive Heart Failure Induced by Myocardial Infarction and Its Relative Mechanism. *Chinese Medicine*.

[49] Soares R. O. S., Losada D. M., Jordani M. C., Évora P., Castro E. S. O. (2019). Ischemia/Reperfusion Injury Revisited: An Overview of the Latest Pharmacological Strategies. *International Journal of Molecular Sciences*.

[50] Toldo S., Mauro A. G., Cutter Z., Abbate A. (2018). Inflammasome, Pyroptosis, and Cytokines in Myocardial Ischemia-Reperfusion Injury. *American Journal of Physiology-Heart and Circulatory Physiology*.

[51] Abd El-Mottaleb N. A., Mahmoud G. S., Negm E. A., Abdel Maksoud F. M. (2019). Garlic Antagonizes Skeletal Muscle Ischemia Reperfusion Injury Through Regulating Inflammation, Apoptosis and Desmin Expression in Adult Male Rats. *International Journal of Physiology, Pathophysiology and Pharmacology*.

[52] Tsai K. L., Hsieh P. L., Chou W. C. (2020). IL-20 Promotes Hypoxia/Reoxygenation-Induced Mitochondrial Dysfunction and Apoptosis in Cardiomyocytes by Upregulating Oxidative Stress by Activating the PKC/NADPH Oxidase Pathway. *Biochimica et Biophysica Acta (BBA) - Molecular Basis of Disease*.

[53] Lei T., Li H., Fang Z. (2014). Polysaccharides From *Angelica sinensis* Alleviate Neuronal Cell Injury Caused by Oxidative Stress. *Neural Regeneration Research*.

[54] Wang J., Cao B., Han D., Sun M., Feng J. (2017). Long Non-Coding RNA H19 Induces Cerebral Ischemia Reperfusion Injury via Activation of Autophagy. *Aging and Disease*.

[55] Zhu M., Chen Q., Liu X. (2014). LncRNA H19/miR-675 Axis Represses Prostate Cancer Metastasis by Targeting TGFBI. *The FEBS Journal*.

[56] Vennin C., Spruyt N., Dahmani F. (2015). H19 Non Coding RNA-Derived miR-675 Enhances Tumorigenesis and Metastasis of Breast Cancer Cells by Downregulating c-Cbl and Cbl-b. *Oncotarget*.

[57] Ling H., Gray C. B., Zambon A. C. (2013). Ca^2+^/Calmodulin-Dependent Protein Kinase II *δ* Mediates Myocardial Ischemia/Reperfusion Injury Through Nuclear Factor-*κ*B. *Circulation Research*.

[58] Thrasivoulou C., Millar M., Ahmed A. (2013). Activation of Intracellular Calcium by Multiple Wnt Ligands and Translocation of *β*-Catenin Into the Nucleus: A Convergent Model of Wnt/Ca^2+^ and Wnt/*β*-Catenin Pathways. *Journal of Biological Chemistry*.

[59] Lehwald N., Tao G. Z., Jang K. Y., Sorkin M., Knoefel W. T., Sylvester K. G. (2011). Wnt-*β*-Catenin Signaling Protects Against Hepatic Ischemia and Reperfusion Injury in Mice. *Gastroenterology*.

[60] Wang S. Q., Li X. J., Qiu H. B. (2014). Anti-Epileptic Effect of Ganoderma Lucidum Polysaccharides by Inhibition of Intracellular Calcium Accumulation and Stimulation of Expression of CaMKII *α* in Epileptic Hippocampal Neurons. *PLoS One*.

[61] Liu L., Sun Y., Wang Y., Xin J., Chen W. (2024). [D-Ala2, D-Leu5]-Enkephalin (DADLE) Provides Protection Against Myocardial Ischemia Reperfusion Injury by Inhibiting Wnt/*β*-Catenin Pathway. *BMC Cardiovascular Disorders*.

[62] Talabattula V. A. N., Morgan P., Frech M. J. (2017). Non-Canonical Pathway Induced by Wnt3a Regulates *β*-Catenin via Pyk2 in Differentiating Human Neural Progenitor Cells. *Biochemical and Biophysical Research Communications*.

[63] You C., Shen F., Yang P. (2024). O-GlcNAcylation Mediates Wnt-Stimulated Bone Formation by Rewiring Aerobic Glycolysis. *EMBO Reports*.

[64] Shu Y., Xiang M., Zhang P. (2018). Wnt-5a Promotes Neural Development and Differentiation by Regulating CDK5 via Ca^2+^/Calpain Pathway. *Cellular Physiology and Biochemistry*.

